# Efficacy of Therapeutic Plasma Exchange in Severe Acute Respiratory Distress Syndrome in COVID-19 Patients from the Western Part of Romania

**DOI:** 10.3390/medicina58121707

**Published:** 2022-11-23

**Authors:** Tamara Mirela Porosnicu, Ciprian Gindac, Sonia Popovici, Adelina Marinescu, Daniel Jipa, Valentina Lazaroiu, Dorel Sandesc, Cristian Oancea, Roxana Folescu, Alexandra-Simona Zamfir, Carmen Lacramioara Zamfir, Laura Alexandra Nussbaum, Ioan Ovidiu Sirbu

**Affiliations:** 1 Center for Complex Network Science “V.Babes”, University of Medicine and Pharmacy, 2 Eftimie Murgu Sq., 300041 Timisoara, Romania; 2 Intensive Care Unit “Pius Branzeu”, Emergency Clinical County Hospital, Liviu Rebreanu 156, 300723 Timisoara, Romania; 3 Discipline of Infectious Diseases, Department XIII, “V.Babes” University of Medicine and Pharmacy, 2 Eftimie Murgu Sq., 300041 Timisoara, Romania; 4 Clinical Hospital of Infectious Diseases and Pneumo Phtisiology “Doctor V.Babes”, Gh.Adam 13 Street, 300310 Timisoara, Romania; 5 Department of Anaesthesia and Intensive Care “V.Babes” University of Medicine and Pharmacy, 2 Eftimie Murgu Sq., 300041 Timisoara, Romania; 6 Center for Research and Innovation in Personalized Respiratory Disease Medicine, “V.Babes” University of Medicine and Pharmacy, 2 Eftimie Murgu Sq., 300041 Timisoara, Romania; 7 Department of Balneology, Medical Recovery and Rheumatology, Family Medicine Discipline, Center for Preventive Medicine for Advanced Research in Cardiovascular Pathology and Hemostaseology, “V.Babes” University of Medicine and Pharmacy, 2 Eftimie Murgu Sq., 300041 Timisoara, Romania; 8 Department of Internal Medicine III, Discipline of Pneumology, “Grigore T. Popa” University of Medicine and Pharmacy, 16 University Str., 700115 Iasi, Romania; 9 Department of Morpho-Functional Sciences I, “Grigore T. Popa” University of Medicine and Pharmacy, 16 University Str., 700115 Iasi, Romania; 10 Department of Neurosciences, “V.Babes” University of Medicine and Pharmacy, 2 Eftimie Murgu Sq., 300041 Timisoara, Romania

**Keywords:** therapeutic plasma exchange, ARDS, inflammatory markers, COVID-19, survival

## Abstract

*Background and Objectives*: The COVID-19 pandemic, caused by the SARS-CoV-2 virus, has surprised the medical world with its devastating effects such as severe acute respiratory distress syndrome (ARDS) and cytokine storm, but also with the scant therapeutic solutions which have proven to be effective against the disease. Therapeutic plasma exchange (TPE) has been proposed from the very beginning as a possible adjuvant treatment in severe cases. Our objective was to analyze the evolution of specific biological markers of the COVID-19 disease before and one day after a therapeutic plasma exchange session, how a change in these parameters influences the patient’s respiratory status, as well as the impact of TPE on the survival rate. *Materials and Methods*: In this retrospective study, we include 65 patients with COVID-19 admitted to the intensive care unit department of our hospital between March 2020 and December 2021, and who received a total of 120 sessions of TPE. *Results*: TPE significantly reduced the following inflammation markers (*p* < 0.001): interleukin-6 (IL-6), C-reactive protein (CRP), lactate dehydrogenase (LDH), fibrinogen, ferritin, and erythrocyte sedimentation rate. This procedure significantly increased the number of lymphocytes and decreased D-dimers levels (*p* = 0.0024). TPE significantly improved the PaO_2_/FiO_2_ ratio (*p* < 0.001) in patients with severe acute respiratory distress syndrome (PaO_2_/FiO_2_ < 100). Survival was improved in intubated patients who received TPE. *Conclusions*: TPE involved the reduction in inflammatory markers in critical patients with COVID-19 disease and the improvement of the PaO_2_/FiO_2_ ratio in patients with severe ARDS and had a potential benefit on the survival of patients with extremely severe COVID-19 disease.

## 1. Introduction

The COVID-19 pandemic, caused by the SARS-CoV-2 virus, has surprised the entire medical world with its devastating effects of severe ARDS [1] and cytokine storm [2], but also by the scant therapeutic solutions which have proven to be effective against the disease. A number of antivirals, [3,4] immunomodulatory [5,6,7], corticosteroid [8], and anticoagulant therapies [9] have aroused interest in treating patients with COVID-19 disease who require intensive therapy, with or without mechanical ventilation. Among the plasma purification techniques, therapeutic plasma exchange (TPE) has been proposed from the very beginning as a possible adjuvant treatment in severe cases requiring admission to intensive care [10]. The rationale behind using TPE for the treatment of COVID-19 patients includes the reduction in inflammatory cytokines levels, the stabilization of the endothelial membrane, treatment of hyperviscosity, reduction in antifibrinolytic mediators, and fibrin degradation products or the elimination of SARS-CoV-2 virus [11]. Therapeutic plasma exchange can eliminate the mediators excessively released in the cytokine storm and improve the biomarkers related to a poor prognosis. These are large molecules (IL-6 = 28 kDa, ferritin = 475 kDa, LDH = 140 kDa, CRP = 25 kDa, D-dimers = 180 kDa, fibrinogen = 340kDa) that can be eliminated by a plasma-filter [12].

The main purpose of this study was to analyze the evolution of specific biological markers in COVID-19 disease before and one day after a TPE session and how the change in these parameters influences the patient’s respiratory status, as well as the impact of TPE on survival rates. All the patients received antiviral, anticoagulant, and corticosteroid treatment.

## 2. Materials and Methods

### 2.1. Experimental Part

Study period: Between March 2020 and December 2021, more than 4500 patients from the western part of Romania who were infected with the SARS-CoV-2 virus were treated in our hospital. The Clinical Hospital of Infectious Diseases and Pneumo-Phthisiology “Doctor V.Babes”, Timisoara, was the main hospital that treated patients with COVID-19 in the western part of Romania. During this period, 535 patients were admitted to the intensive care unit (ICU) with severe forms of the disease, out of which over 401 patients required intubation and mechanical ventilation. Patients admitted to the ICU during this period presented severe lung damage (moderate or severe ARDS). Therapeutic plasma exchange was initiated in 65 adult patients who experienced a severe impairment of respiratory status or an increase in specific biological markers (IL-6, ferritin, ESR, D-dimers, LDH, CRP, and fibrinogen) for excessive cytokine release syndrome as a complication of COVID-19.

Ethical aspects: The study was conducted in the ICU department and the written informed consent was obtained from all study participants. The Ethical Committee of the hospital (nr. 13037/24/12/2021) approved the study.

Inclusion criteria in patient selection were that patients were aged over 18 years, had moderate or severe ARDS and/or cytokine storm (exaggerated increase in specific parameters for systemic inflammation), in addition to the availability of clinical and laboratory data before and at one day after conducting TPE sessions. Our patients had clinical respiratory deterioration requiring admission to the intensive care unit and needed increased oxygen (more than 30 L/min), SpO_2_ < 90%, respiratory rate over 30 breaths/min, and PaO_2_/FiO_2_ < 200 mmHg (moderate ARDS 100 ≤ PaO_2_/FiO_2_ < 200 or severe ARDS = PaO_2_/FiO_2_ < 100).

Data collection: A series of demographic data were analyzed: age, sex, BMI, respiratory status, number of days from the beginning of COVID-19 symptoms until the first TPE, number of sessions of TPE, and comorbidities (diabetes, hypertension, obesity, and COPD).

Variables: The following parameters were included for the analysis: biological markers for inflammation (IL-6, CRP, ferritin, D-dimers, ESR, LDH, procalcitonin, and fibrinogen). Leukocyte count, lymphocytes count and percentage, hemodynamic parameters, vasopressor requirement, and temperature were also analyzed.

To evaluate the impact of organ failure after the TPE session, we investigated liver enzymes, blood urea nitrogen (BUN), creatinine, pH, lactate, SOFA, and APACHE II scores. Oxygenated status of the patients was examined using the following: PaO_2_/FiO_2_, respiratory rate, ROXINDEX score, HACOR score, and the oxygenation index (OI).

The TPE sessions were performed using the Prismaflex (Baxter International, Deerfield, IL, USA) device with a TPE 2000 plasma-filter or by using the HF404 machine (Infomed) with a Granopen 60 plasma-filter (LF 060-00). As a substitute, 5% human albumin solution or fresh frozen plasma were used in doses of 1–1.3 times the patient’s plasma volume. Fractionated heparin and/or citrate were used as an anticoagulant [13]. We did not use convalescent plasma for these patients.

The determination of biochemical parameters was performed at a hospital laboratory with the COBAS INTEGRA 400 plus device (Roche diagnostics) and the reverse transcription polymerase chain reaction (RT-PCR) for SARS-CoV-2 with a BIONEER extractor and EXICYCLER 96 amplifier (Bioneer, Daejeon, Republic of Korea). COVID-19 case confirmation was obtained using the CFX96 Real-Time PCR Systems (Bio-Rad, Hercules, CA, USA). The viral RNA was extracted with the NIMBUS extractor, using the STARMag 96X4 Universal Cartridge Kit (Seegene, Seoul, Republic of Korea).

### 2.2. Statistical Analysis

A data analysis was performed using the Statistical Package for Social Sciences v.25 (IBM SPSS Statistics, Chicago, IL, USA). A p value of less than 0.05 was considered statistically significant. Paired sample Wilcoxon tests were used for the statistical analysis. Due to the small sample size of the data set, we prefer this test for our data. There were a few huge values for some covariates (such as IL6 over 5000 pg/mL), which is why Wilcoxon’s test may be more appropriate. Quantitative variables were tested for normal distribution and compared by means of a Wilcoxon test paired sample.

## 3. Results

We analyzed data from 65 adult patients and 120 TPE procedures: 41 patients with 1 session, 13 patients with 2 sessions, and 11 patients with 3 or more sessions (2 with 3 sessions, 4 with 4 sessions, 1 with 5 sessions, 2 with 6 sessions, and 2 with 7 sessions) (Table 1).

Laboratory and clinical data of patients with COVID-19 were analyzed one day before and one day after TPE and using the Wilcoxon test paired sample (Table 2). These 65 patients had moderate or severe ARDS (PaO_2_/FiO_2_ median was 98.25) or an increased inflammatory marker [14].

A lot of covariates were analyzed before and after 120 TPE sessions for 65 patients. There were only 110 sessions to evaluate the PaO_2_/FiO_2_ ratio after TPE. We excluded data from 10 sessions for patients on ECMO (Table 1). This section may be divided by subheadings. It should provide a concise and precise description of the experimental results, their interpretation, as well as the experimental conclusions that can be drawn.

Large molecules such as ferritin, D-dimers, CRP (C-reactive protein), fibrinogen, LDH (lactate dehydrogenase), and IL-6 were eliminated by TPE in most of the cases with a strong statistical significance, as we expected (*p* < 0.05, Table 2). In our group, the average values for these markers before the first TPE session are: IL-6 = 799 pg/mL, ferritin = 2364 µg/L, D-dimers = 5.18 µg/mL, CRP = 122 mg/L, LDH = 579 U/L, and fibrinogen = 4.90 g/L) (Table 2).

**Figure 1:**
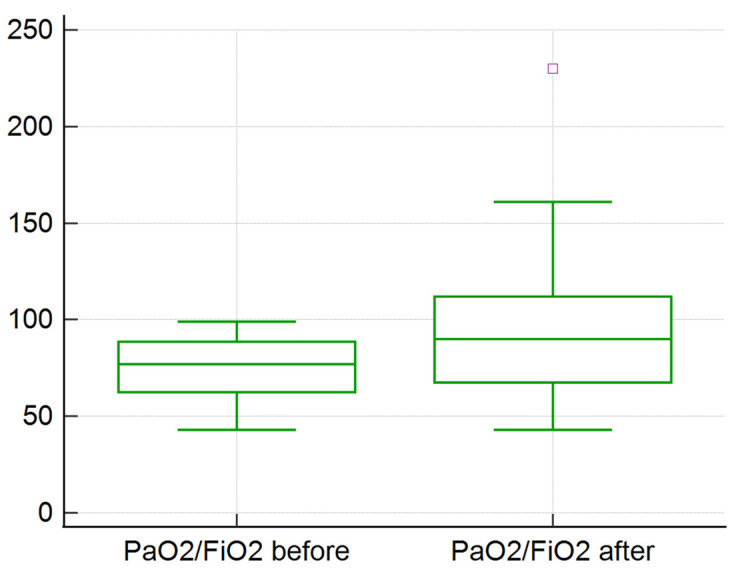
TPE impact on the PaO_2_/FiO_2_ ratio in patients with severe ARDS (55 sessions).

**Figure 2:**
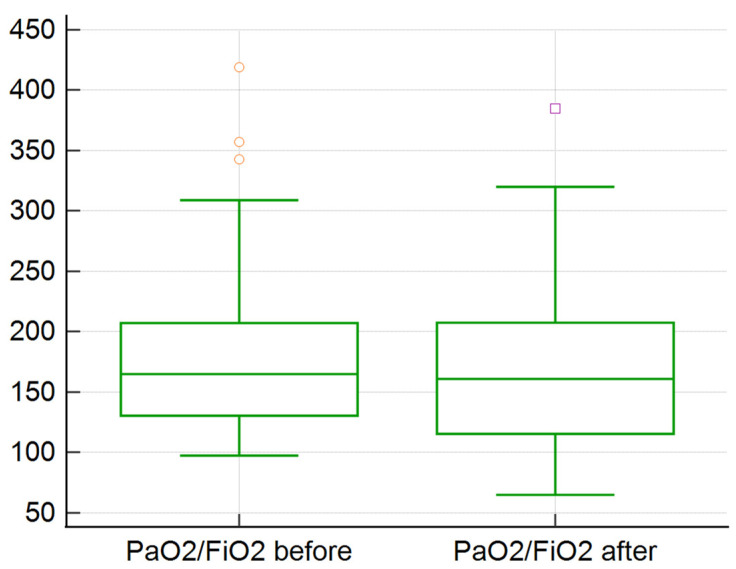
TPE impact on PaO_2_/FiO_2_ ratio in patients with non-severe ARDS (55 sessions).

There was no statistically significant improvement in the following studied clinical features: pulmonary status (respiratory rate, PaO_2_/FiO_2_, OI), hemodynamic parameters (TAM, AV, and need for vasopressor agents), organ functionality (BUN, creatinine, lactate, SOFA and APACHE II score), none of which were improved (*p* > 0.5). We observed that the median lymphocyte percentage or the absolute count improved after TPE procedure. A particular group with severe ARDS (PaO_2_/FiO_2_ < 100) had a statistically significant improvement in oxygenation after TPE (*p* = 0.002) (Figure 1). The rest of the patients with mild/moderate ARDS had no statistically significant improvement in oxygenation after TPE sessions (*p* = 0.17) (Figure 2). The mean value for PaO_2_/FiO_2_ in the group with severe ARDS increased from 75 to 92; the mean decreased from in the rest of the group 184 to 173 (Figure 1 and Figure 2).

A total of 61% of the patients included in the study were mechanically ventilated at the time of the first TPE. The oxygenation index (OI) was 21.56 before TPE and 21.8 after TPE and four patients needed ECMO (where the P/F ratio has no reason to be evaluated). There were only 16 survivors in our 65-patient group after 28 days (24.61%). Between 40 mechanical ventilated patients, there were 7 survivors. In the subgroup of 11 patients with more than 2 TPE procedures/patient (average 4.8), the P/F ratio remained at the same: the median value is 121 before TPE versus 122 after TPE, and an average of 144 versus 147. There was no statistically significant impact on the P/F ratio (*p* = 0.36). More than two TPE sessions for one patient had no statistically significant benefit in oxygenation in our study. In 63 of the TPE sessions, we have observed an improvement in P/F ratio, whereas in the other 47 sessions, no improvement after TPE was shown. In the group with severe ARDS in 55 TPE sessions, 41 resulted in an improvement in oxygenation and in the other group with moderate /mild ARDS in 55 sessions, only 22 had an improvement in oxygenation.

**Figure 3:**
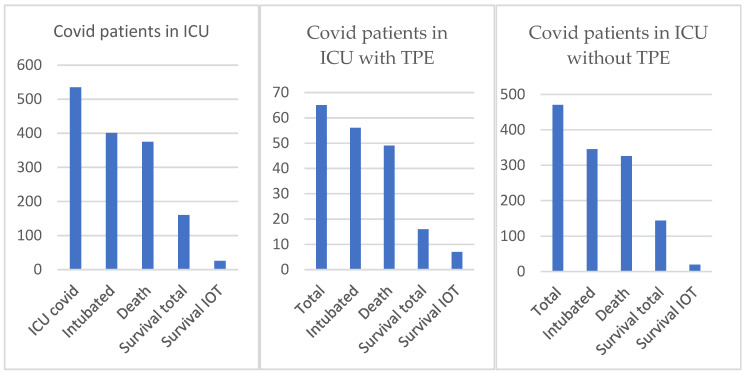
Survival in ICU: total COVID patients versus COVID patients with/without TPE.

From a total number of 535 patients admitted to ICU, 401 were finally invasively mechanical ventilated, and 375 died and 160 survived. Only 26 from the intubated patients (6.5%) survived. From a total of 65 patients who performed TPE, 56 were invasively mechanical ventilated from the beginning; a total of 16 of them survived and 7 from the intubated patients (12.5%). There was an improvement in the survival rate (*p* = 0.05) between patients with invasive mechanical ventilation and who had TPE performed on them compared to the survival rate of the patients with invasive mechanical ventilation without TPE (5.2%) in our ICU department along this period of COVID-19 disease (Figure 3).

## 4. Discussion

The SARS-CoV-2 virus had a global impact and there are still no clear treatments for moderate and severe ARDS in COVID-19 patients. More than 50% of patients with cytokine storm develop ARDS and early recognition and control of dysregulated immune response are essential [15].

Our study showed that TPE significantly reduced the levels of the main inflammatory biomarkers associated with a poor prognosis. Therapeutic plasma exchange had a positive impact on the respiratory status (improvement in PaO_2_/FiO_2_) in patients with a P/F ratio under 100. Moreover, we also observed slight improvements in patients with a P/F ratio over 100. The impact of TPE in the COVID-19 critically ill patient was not significant regarding the case of end-organ failure.

The first extrarenal clearance procedures were tried in patients with COVID-19 in Asia [16]. Case series and individual reports have shown the effectiveness of TPE in the reduction in inflammation markers [16]. The results seemed very encouraging and suggested both the overall reduction in inflammation markers and the improvement of respiratory parameters: reduction in oxygen demand, decrease in respiratory rate, and early extubating [16]. With the outbreak of the pandemic, it has been observed that there is a link between the severity of the disease following infection with the SARS-CoV-2 virus and a series of biological markers whose values are altered. Changes in these markers (IL-6, IL-1, TNFα, D-dimers, CRP, ferritin, LDH, and fibrinogen), especially inflammatory ones, have a specificity for a poor prognosis [17]. Subsequently, attempts were made to demonstrate the reduction in inflammation markers by a series of plasma purification procedures, by the administration of immunomodulatory agents, and with the use of anti-inflammatory drugs. Out of these options, TPE significantly reduced specific markers in the case of cytokine storm (CS) but had less impact on respiratory parameters or survival rate [18].

There have been few studies in the literature that have analyzed the data of patients receiving TPE and patients receiving only standard therapy [13,19,20]. Thus, Fahad Faqihi et al. [19], in a randomized study, analyzed 43 patients who received standard treatment plus TPE and 44 patients who received only standard treatment and observed that mortality at 35 days in the group who received TPE compared to the control group was not significantly lower (20.9% vs. 34.1%), *p*-value 0.58. However, biological markers of inflammation and acute phase were significantly reduced in the TPE group, and thus it was concluded that in the TPE group, patients had a faster clinical recovery [19]. This suggests that although all markers of inflammation are significantly reduced by TPE, the final results are not the desired ones. In the case of COVID-19 critically ill patients, the implementation of TPE under the mentioned circumstances could not restore the damaged pulmonary tissue and did not have a major impact on the improvement of other organ function. In patients with life-threatening COVID-19, TPE added to standard therapy compared with standard therapy alone resulted in clinical recovery but did not affect 35-day mortality, MSOF score, or the P/F ratio [19]. One of the limitations of our study is that although it includes a large number of patients, we did not use a control group, so we could not analyze the differences between the days of hospitalization and mortality.

Sultan Mehmood Kamran et al. [20] analyzed 45 patients who received TPE and 45 patients who did not receive TPE and found that the survival rate was higher in patients in whom TPE was initiated in the first 12 days after the onset of symptoms. They also observed that the duration of hospitalization was reduced in the group with TPE (10 days versus 15 days), and the mortality was higher (17.9%) in the group where TPE was performed later than 12 days after the onset of symptoms [20]. In our study, we observed that the average from the onset of symptoms until TPE was performed is 16.6 days. Thus, we can conclude that if TPE had been done earlier, the survival rate maybe would have been higher. This was difficult to achieve due to the epidemiological conditions and the large number of patients who needed admission in the hospital or in a place in ICU, in addition to late presentation to the hospital and to ICU.

Faryal Khamis et al. analyzed 31 patients with a mean age of 51 years, of whom 90% were men, 11 received TPE in the first two weeks of the disease, and 20 patients represented the control group [13]. They observed that in the TPE group mortality was significantly lower and the rate of extubating of patients was much higher (73% versus 20%), but the duration of hospitalization in intensive care was longer, with 14 days versus 6 days [13].

Cytokine storm is an aggressive inflammatory response acting through the release of an excessive amount of proinflammatory products [21]. It is correlated with interferon antagonism which inhibits the innate immune response, and it is directly proportional to lung injury, MSOF, and mortality [21].

In seven patients who had comorbidities such as hypertension (14.3%), asthma (14.3%), and diabetes (14.3%), Ikram Zaid et al. [22] obtained statistically significant results in the reduction in inflammatory markers and acute phase reactants (IL-6 *p*-value = 0.0004, fibrinogen *p*-value = 0.015, ferritin *p*-value = 0.011, and PCR *p*-value = 0.06), and all seven patients survived [22]. Our study clearly demonstrates a statistically significant decrease in biological markers (*p*-value: IL-6 < 0.0003, ferritin < 0.0001, fibrinogen < 0.0001, CRP = 0.0001, D-dimers = 0.0024, LDH < 0.001, and ESR < 0.0001), but the survival rate was much lower due to the large number of critical patients with more severe inflammatory markers and acute phase reactants compared with this study. The patients who received TPE in our hospital had a more critical situation. In our group, the average values for these markers before the first TPE session are even higher: IL-6 = 799 pg/mL, ferritin = 2364 µg/L, D-dimers = 5.18 µg/mL, CRP = 122 mg/L, LDH = 579 U/L, and fibrinogen = 4.90 g/L. We observed that using the TPE procedure in these cases can be a rescue procedure for the patients with severe ARDS forms (median P/F ratio 98.2).

Elimination of LDH is of particular importance, as its increase can be harmful by negatively impacting lactate levels, the activation of cytokines being an important marker of severity in the disease [23]. The same can be said for ferritin, IL-6 or CRP, as their increase plays a key role in inflammation [24].

Our study was conducted to evaluate the efficacy of TPE in patients with COVID-19 disease because critically ill patients had a high mortality rate due to the development of severe inflammatory syndrome and organ failure. Thus, Seyed Mohammad Reza Hashemian et al. [25] in a study of 15 patients (9 patients were male, and 6 patients were female), observed the efficacy of TPE by analyzing inflammatory cytokines, the PaO_2_/FiO_2_ ratio, and acute phase proteins. The ratio of biological markers in their study was significantly improved after the TPE session, where 9 (60%) of the 15 patients survived and 6 (40%) patients who needed mechanical ventilation died [25]. In our study of 65 patients (45 were male, and 20 were female), we found that the survival rate was 24.6% (16 patients) and the rest of the patients who died developed multiple organ failure.

The plasma purification technique used in the studies published in the literature [13,20,26] was not different in terms of the volumes used or filters; the only thing that was used in various combinations was the substitution solution: albumin 5% and saline 0.9% [25], FFP [13,22], FFP and saline solution 0.9% [20], 5% albumin, and FFP [19], and no adverse reactions were observed to aggravate the clinical condition of the patients [13,19,20,22]. No adverse reactions were observed that aggravated the clinical condition of the patients [13,19,20,22]. No adverse reactions were observed in our study. We consider that TPE is a safe procedure in terms of side effects, and we have no reservations in initiating it from this point of view. On the other hand, in the worst case scenario, we can consider it a cosmetic maneuver to purify the patient’s plasma, which is a breath of oxygen in the fight against COVID-19 disease. We consider that a favorable evolution in the critically ill patient with COVID-19 disease could be influenced even after the beginning of cytokine storm by associating other treatment options [26,27,28] such as antivirals, anti-inflammatory, immunomodulatory drugs, and anticoagulation [4,7,9], and by avoiding self-induced lung injury (SILI) by early intubation, followed by optimal protective mechanical ventilation [29,30]. The lung parenchyma compromised by inflammation and fibrosis cannot be restored by plasma clearance, but further damage might be stopped. TPE may be effective for reducing the systemic inflammation that could be involved in worsening the organ functions and may improve the outcome in patients with ARDS if it is started early [20].

A limitation of the study is that it is a retrospective uncontrolled single-center study, and we did not have a control group to analyze the difference between mortality and the number of days of hospitalization.

## 5. Conclusions

TPE can be used to reduce inflammation markers in COVID-19 critically ill patients and improve the PaO_2_/FiO_2_ ratio in patients with severe ARDS. This procedure also showed a minimum benefit in the survival of patients with extremely severe forms of COVID-19 disease. TPE should be used early with critically ill patients with ARDS. We consider that TPE and many other therapeutic approaches represent distinct pieces from the complicated puzzle of COVID-19 which still remains to be solved.

## Figures and Tables

**Table 1:** Demographic and baseline characteristics of critical patients with COVID-19 (n = 65).

Age-mean (min–max)	52.70 (21–80)
Body Mass Index-mean	33.2
Gender	
Male	45
Female	20
Days from RT-PCR COVID-19 and TPE mean, (min–max)	16.6 (5–51)
Respiratory status	
Invasive Mechanical Ventilation	40
Noninvasive Mechanical Ventilation	25
Comorbidities (no. and % from total no. of patients)	
Diabetes Mellitus	10 (15.38%)
Hypertension	33 (50.76%)
Obesity	27 (41.53%)
COPD/asthma	6 (9.23%)
No. of sessions (total)	120
Patients with 1 session	41
Patients with 2 sessions	13
Patients with more than 2 sessions	11

RT-PCR: reverse transcription polymerase chain reaction; COPD: chronic obstructive pulmonary disease.

**Table 2:** Average and medium values of analyzed biological markers, before and after TPE, *p*-value. (n = 65).

Variable	Average/Median before TPE	Average/Median after TPE	*p*-Value for Median
IL-6, pg/ml	799/129	480/79	0.0003
Ferritin, µg/L	2364/1529	1660/1120	<0.0001
D-dimers, µg/ml	5.18/1.9	3.50/1.57	0.0024
CRP, mg/L	122/88	87/60	0.0001
LDH, U/L	579/512	461/419	<0.0001
PCT, ng/ml	2.28/0.33	2.26/0.42	0.34
Fibrinogen, g/L	4.90/4.23	3.42/3.26	<0.0001
ESR, mm/h	46/35	22/15	<0.0001
Leucocytes, ×10^3^/µL	14/13	16/15.5	0.19
% Lymphocytes	7.66/5.2	8.14/5.5	0.53
Lymph abs, ×10^3^/µL	0.96/0.69	1.11/0.80	0.003
TAM, mmHg	80.9/77	81.9/80	0.9
Temperature, °C	36.5/36.4	36.6/36.4	0.26
BUN, mg/dL	72.7/58.5	74.2/62	0.29
Creatinine, mg/dL	1.02/0.8	1.08/0.81	0.98
PH	7.42/7.43	7.41/7.42	0.79
Lactate, mmol/L	2.49/2.30	2.55/2.27	0.72
SOFA	7.71/7	7.76/7	0.96
APACHE 2	11.7/11	12.1/12	0.056
PaO_2_/FiO_2_	**128.1/98.25**	**131.7/113.25**	0.23
**P/F < 100 for 55 sessions**	**75/77**	**92/90**	**0.0002**
P/F ≥ 100 for 55 sessions	184/168	173/162	0.17

IL-6: intrleukin-6; CRP-C reactive protein; LDH: lactate dehydrogenase; PCT: procalcitonin; ESR: erythrocyte sedimentation rate; TAM-mean arterial pressure; BUN: blood urea nitrogen; SOFA: Sequential Organ Failure assessment; APACHE 2: Acute Physiology and Chronic Health Evaluation; PaO_2_/FiO_2_: pressure of arterial oxygen to fractional inspired oxygen concentration; P/F: pressure of arterial oxygen to fractional inspired oxygen concentration; TPE: therapeutic plasma exchange.

## Data Availability

Not applicable here.
